# Structural Design of a Special Machine Tool for Internal Cylindrical Ultrasonic-Assisted Electrochemical Grinding

**DOI:** 10.3390/mi14010222

**Published:** 2023-01-15

**Authors:** Xiaosan Ma, Feng Jiao, Wenbo Bie, Ying Niu, Shuaizhen Chu, Zhanzhan Hu, Xiaohong Yang

**Affiliations:** 1 School of Mechanical and Power Engineering, Henan Polytechnic University, Jiaozuo 454000, China; 2 College of Mechanical and Electronic Engineering, Huanghe Jiaotong University, Jiaozuo 454950, China; 3 School of Electrical and Mechanical Engineering, Pingdingshan University, Pingdingshan 467000, China

**Keywords:** internal cylindrical ultrasonic-assisted electrochemical grinding, special machine tool, workpiece clamping and rotating device, ultrasonic vibration system, conductive grinding wheel dressing

## Abstract

During the process of internal cylindrical ultrasonic-assisted electrochemical grinding (ICUAECG), both the workpiece and the conductive grinding wheel are rotating, the machining space is closed and narrow, the electrolyte is difficult to spray into the machining area, and the insulation between the workpiece and the machine bed is challenging. According to the machining characteristics of ICUAECG, the structure of a special machine tool was designed to mitigate these problems. In particular, the rotation, electrolyte supply, electric connection, and insulation modes of the workpiece clamping parts were studied, yielding a novel workpiece clamping- and rotating-device design. This structure can fully use the internal space of the hollow spindle of the machine tool, effectively reduce the external moving parts, and achieve the appropriate liquid injection angle of the electrolyte. The ultrasonic vibration system and its installation mechanism, the dressing device of the conductive grinding wheel, and the electric grinding spindle-mounting and -fixing device were analyzed in detail. Then, a special machine tool for ICUAECG was designed, the operability and feasibility of which were verified by experiments involving conductive grinding wheel dressing and ICUAECG.

## 1. Introduction

Electrochemical grinding (ECG) is a hybridized grinding technology combining electrochemical machining (ECM) with conventional mechanical grinding [1,2]. Its advantages, namely, high machining efficiency, small grinding force, good surface quality, and minor wheel wear, substantiate its wide applications in the precision and ultraprecision machining of difficult-to-machine materials such as stainless steels, titanium alloys, superalloys, and metal matrix composites. Ultrasonic-assisted electrochemical grinding (UAECG) is a novel hybridized electrochemical grinding technology developed by combining ultrasonic vibration-assisted machining [3,4] and electrochemical grinding [5,6]. Compared with ordinary ECG, the UAECG process is more stable [7], the grinding force is smaller [5,7], the conductive grinding wheel wear is alleviated [5], and the machined surface roughness is reduced [5,7,8,9]. Therefore, with high-end equipment and precision machinery technology development, UAECG is receiving increasing attention and application in industry.

The theoretical research and application promotion of any processing technology can only occur with the research and development of special equipment. After Keeleric introduced ECG technology in 1952, considerable research on the design and development of ECG and UAECG special machine tools was conducted. Sonia [1] developed simple ECG equipment that processed mild steel and copper workpiece with NaCl and CuSO_4_ aqueous solution as electrolytes. Bhuyan et al. [10] designed a tabletop ECG setup and conducted ECG experiments on aluminum workpieces with NaOH aqueous solution as the electrolyte. Wang et al. [11,12] developed a face ECG machine tool for the precision machining of toroidal surfaces. During the machining process, the electric field distortion on both sides of the machining surface is effectively avoided by using the grinding head to make a circular translation feed so that the machining surface shape error can be controlled within 5 μm and the surface roughness parameter Ra can reach a value of 0.21 μm.

Aiming at the automatic control of ECG machine tools, Xiao et al. [13] developed the NC-embedded PC open integrated control system of ECG with the hardware structure of the industrial tablet computer as the human–computer interaction platform, taking a multifunctional motion control card and multifunctional data acquisition card as the control core and realizing the human–computer interaction, automatic control, monitoring and protection functions of the processing process. Gan et al. [14,15,16,17] developed 4- and 5-axis CNC machines and a 5-axis automatic programming system for the ECG of complex structural parts such as engine blades and profiles.

For UAECG, Wu et al. [5,7,18] developed an ultrasonic-assisted electrochemical mill-grinding vertical machine tool by installing an ultrasonic electric spindle on a 3-axis NC machine tool. They studied the planar ultrasonic-assisted electrochemical mill-grinding of Ti-6Al-4V with a metal-bonded CBN grinding wheel of 1.8 mm in diameter. Liu et al. [8,9] developed an ultrasonic-assisted electrochemical drill-grinding machine tool for dealing with the precision machining of small holes with a diameter of 1.8 mm. They studied the ultrasonic-assisted electrochemical drill-grinding of 304 stainless steel plates with a thickness of 0.5 mm using metal bonded diamond ball head grinding tools.

The above achievements in the design and development of ECG and UAECG machine tools were mainly related to planar grinding, small hole drill-grinding, curved surface mill-grinding, etc. In the above cases, the feed motion mode was relatively simple during the machining process, and the workpiece was in a static state. Most developed UAECG machine tools had quite expensive ultrasonic electric spindles with a small ultrasonic vibration power output, so only smaller conductive grinding wheels could be used. However, to the best of the authors’ knowledge, no research results have been reported either on the cylindrical and internal cylindrical ECG with rotating grinding wheel and workpiece, or on UAECG suitable for a large-size conductive grinding wheel and ordinary grinding electric spindles, or on ICUAECG with a relatively small machining space.

With the development of grinding technology, more and more 4- and 5-axis CNC machines and regular machine tools have been applied to hybridized grinding [14,15,16,17] and super abrasive machining [19,20] after a certain degree of transformation. Therefore, for ICUAECG, its special machine tool can be transformed from a regular machine tool.

This study attempts to fill the above gap by designing the structure of a special machine tool for the ICUAECG, following its main principles and characteristics. In particular, the key issues of rotation, electrolyte supply, electricity connection and insulation of the workpiece clamping parts were examined, and a new workpiece rotating clamping device was developed. At the same time, the structure of the ultrasonic vibration system, the dressing device of the conductive grinding wheel, and the mounting mechanism of the grinding electric spindle suitable for large conductive grinding wheels and ordinary grinding electric spindles were designed and studied. Finally, experiments on conductive grinding wheel dressing and ICUAECG were performed, verifying the rationality of the proposed special machine tool structure.

## 2. Machining Principle and Characteristics of ICUAECG

**Figure 1:**
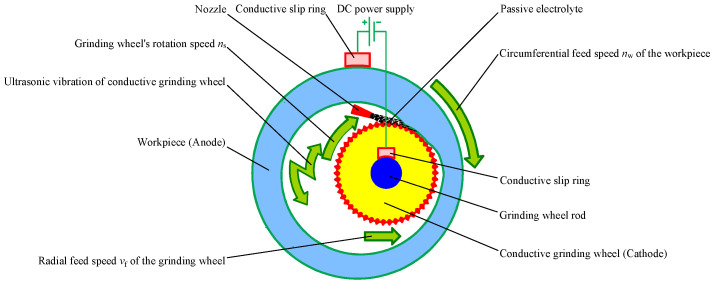
Schematic diagram of the ICUAECG.

Figure 1 shows a schematic diagram of the ICUAECG process. The conductive grinding wheel is installed on the horn and connected to the ultrasonic vibration system. The workpiece and the conductive grinding wheel are connected with the positive and negative poles of the DC power supply through electric brushes (or conductive slip rings), respectively. During machining, the conductive grinding wheel rotates at a rotation speed of *n*_s_ and feeds along the radial or axial direction at a feed rate of *v*_f_. At the same time, the workpiece rotates at a rotation speed of *n*_w_ to realize circumferential feeding. The conductive grinding wheel contacts the inner hole surface of the workpiece through the abrasive particles on its surface. A small interelectrode gap is formed between the workpiece and the metal bond of the conductive grinding wheel. The passive electrolyte is sprayed into the interelectrode gap through the nozzle. Under the action of the electrolytic current, the surface material of the workpiece is electrochemically dissolved and then forms a passive film, which hinders or reduces the continuous progress of the electrochemical anode dissolution. Its hardness is significantly lower than that of the workpiece substrate material. When the workpiece continues to feed along the rotation direction, and the grinding wheel continues to feed along the radial or axial direction, the passive film is scraped off by the abrasive particles on the surface of the conductive grinding wheel, and the electrochemical anode dissolution can continue. With the circulation of electrochemical anode dissolution, passive film generation, and passive film scraping, the surface material of the inner hole of the workpiece is continuously removed until the specified dimension requirements are met. In the machining process, high-frequency ultrasonic vibration is exerted on the conductive grinding wheel, which strongly impacts the workpiece’s material removal and surface generation mechanism to improve the stability, machining efficiency, and surface accuracy of ECG, as well as reducing the wear on the grinding wheel.

Different to ordinary internal cylindrical grinding, ICUAECG has the following specific features: (1) the workpiece and the conductive grinding wheel need to be connected with the positive and negative poles of the DC power supply, respectively, and the circuit connection device should be equipped and the lead wire should be appropriately arranged; (2) the workpiece and grinding wheel must be well insulated from the machine bed to prevent electric leakage; (3) the angle and direction of electrolyte spraying significantly influence the uniformity of electrolyte distribution in the machining area, the stability of the electrolytic reaction process and the machined surface quality; (4) the electrolyte is corrosive to the machine bed, so it is necessary to collect the electrolyte and prevent splashing; (5) the conductive grinding wheel needs to be connected with the ultrasonic vibration system, and the horn is the grinding wheel rod. The shapes and dimensions of the conductive grinding wheel and the horn have essential effects on the vibration performance of the ultrasonic vibration system.

In short, the ICUAECG differs from other forms of UAECG in the following two characteristics: (1) both the workpiece and the grinding wheel are in rotary motion; (2) the machining area is a closed, narrow, and small space.

The above characteristics make the special machine tool for ICUAECG more complex than ordinary internal cylindrical grinding and other forms of UAECG.

## 3. Structural Design of Special Machine Tool

### 3.1. The Overall Structure

**Figure 2:**
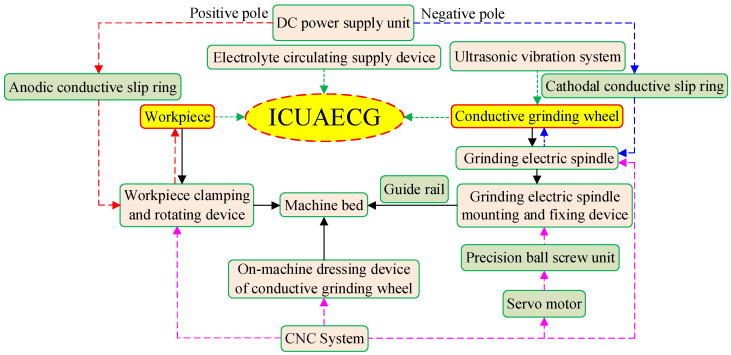
Main components of the ICUAECG machine tool.

Like the ordinary internal cylindrical grinding machine tool, the ICUAECG machine tool mainly consists of the machine bed, workpiece clamping and rotating device, a grinding electric spindle mounting and fixing device, etc. In addition, according to the requirements of ultrasonic vibration and ECG, the electrolyte circulating supply device, DC power supply device, on-machine dressing device of the conductive grinding wheel, numerical control system, ultrasonic vibration system, and other devices need to be equipped. The main components of the ICUAECG machine tool are depicted in Figure 2.

According to their structural features and machining modes, machine tools are classified into vertical and horizontal. In previous research on UAECG [5,7,8,9,18], very small lightweight conductive grinding wheels and integrated ultrasonic electric spindles were used; the conductive grinding wheel on-machine dressing was not considered, and all machine tools were all vertical structures. For the ICUAECG, a large conductive grinding wheel and ordinary grinding electric spindle are used, the ultrasonic vibration system needs to be installed, the volume and weight of the grinding electric spindle and conductive grinding wheel are large, and considering the conductive grinding wheel on-machine dressing, the horizontal structure is appropriate for the special machine tool.

**Figure 3:**
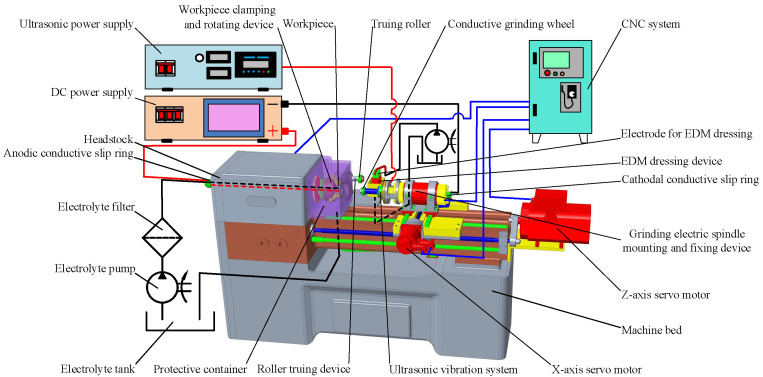
Overall structure of the special machine tool for ICUAECG.

According to the above analysis, the overall structure of the special machine tool for ICUAECG was designed, as shown in Figure 3.

The electrolyte-circulating supply device comprises an electrolyte tank, an electrolyte pump, a filter, an electrolyte supply pipe, and a liquid nozzle. The electrolytic power supply device is composed of an electrolytic DC power supply, anodic conductive slip ring, cathodal conductive slip ring, connecting wire, etc. The electrolyte supply pipe and the connecting wire are installed on the outer ring of the anodic conductive slip ring pass through the inner part of the workpiece clamping and rotating device, as shown by the black and red dotted lines in Figure 3.

During the ICUAECG process, under the control of the NC system, the grinding electric spindle drives the conductive grinding wheel to rotate at the specified speed; the servo motors drive the grinding electric spindle mounting and fixing device to feed along the X- or Z-axes, and the machine tool hollow spindle drives the workpiece clamping and rotating device to rotate to achieve the circumferential feed of the workpiece. The ultrasonic electrical signal and energy output by the ultrasonic power supply are converted into the ultrasonic mechanical vibration of the conductive grinding wheel through the ultrasonic vibration system. The conductive grinding wheel is connected with the negative pole of the DC power supply through the cathodal conductive slip ring at the tail of the grinding electric spindle. The electrolyte pump transmits the electrolyte to the machining area through the filter and electrolyte supply pipe. The protective container is used to prevent electrolyte splashing during machining, collect the electrolyte and transport it to the electrolyte tank through the return pipeline.

When dressing the conductive grinding wheel, the roller truing device can be used for truing. Then the reverse electrolysis method can be used for dressing, or the EDM dressing device can be used for EDM dressing the conductive grinding wheel.

### 3.2. Workpiece Clamping and Rotating Device

In the special machine tool for ICUAECG, the workpiece clamping and rotating device should meet the following requirements:

(1) It should clamp the workpiece to rotate with the hollow spindle of the machine tool;
(2) The workpiece should be connected with the positive pole of the DC power supply through the anodic conductive slip ring;
(3) It should provide electric insulation of the workpiece from the machine bed;
(4) It should support the electrolyte supply pipeline inside the hollow spindle of the machine tool and ensure that the electrolyte supply pipeline remains fixed when the hollow spindle of the machine tool rotates;
(5) The device should be reliably fixed to the hollow spindle of the machine tool.

**Figure 4:**
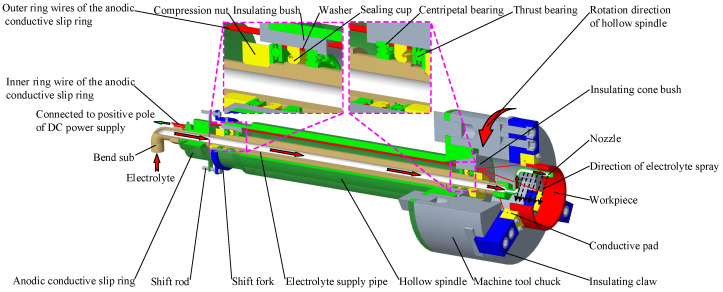
Schematic diagram of the workpiece clamping and rotating device.

According to the above requirements, the workpiece clamping and rotating device design is shown in Figure 4. The device is mainly composed of the hollow spindle of the machine tool, electrolyte supply pipe, anodic conductive slip ring, insulating bush, insulating cone bush, nozzle, centripetal bearing, thrust bearing, sealing cup, shift fork, shift rod, and other parts. The insulating bushing, insulating cone bush made of nylon material, and centripetal bearing are used to support the electrolyte supply pipe installed in the hollow spindle to ensure good insulation between the electrolyte supply pipe and the hollow spindle to prevent the positive electricity of the workpiece from being transmitted to the machine bed through the electrolyte, electrolyte supply pipe, or hollow spindle during machining. The compression nuts and thrust bearings at both ends axially fasten the electrolyte supply pipe inside the hollow spindle and can flexibly rotate. The sealing cup prevents electrolyte and external dust from entering the hollow spindle. The inner ring of the anodic conductive slip ring is sleeved on the electrolyte supply pipe and fastened by the jackscrew. The shift fork and rod mounted on the hollow spindle are used to drive the outer ring of the anodic conductive slip ring to rotate with the hollow spindle. The inner ring wire of the anodic conductive slip ring is connected with the positive pole of the DC power supply. The outer ring wires of the anodic conductive slip ring pass through the wire-threading holes on the insulating bush and insulating cone bush and are connected with the conductive pads fixed on the insulating claws through the wiring screws. The insulating claws and conductive pads on the machine tool chuck are used to clamp and loosen the workpiece and ensure insulation between the workpiece and the machine body. The electrolyte nozzle is curved to avoid movement interference with the conductive grinding wheel during machining. The cylindrical surface on the side of the electrolyte nozzle is machined with a long and narrow electrolyte outlet to ensure that the electrolyte can be sprayed into the machining area along the direction shown in Figure 4, to reduce the electrolyte splash.

In the ICUAECG process, the hollow spindle drives the workpiece to rotate through the machine tool chuck and insulating claws. The outer ring and the outer ring wires of the anodic conductive slip ring rotate along the direction shown in the figure through the shift fork and rod. The inner ring and the inner ring wires of the anodic conductive slip ring, the electrolyte supply pipe, and the electrolyte nozzle remain fixed to ensure the normal operation of the electrolyte supply, spray, and positive pole of the DC power supply.

The hollow spindle is installed in the headstock of the machine tool through bearings and driven by a three-phase AC asynchronous motor. The CNC system controls their rotational speed through a frequency converter to realize the workpiece circumferential feed rate adjustment during machining.

In this workpiece clamping and rotating device, the electrolyte supply pipeline, nozzle, and positive power supply wires of the DC power supply are all installed inside the hollow spindle and chuck of the machine tool. On the premise of ensuring the machine tool’s regular operation, the hollow spindle’s internal space is fully utilized; external suspension devices such as external nozzles and connecting lines are reduced, and the volume is reduced. At the same time, the device uses a long and narrow electrolyte outlet on the nozzle side, and the electrolyte spray direction faces the machining area. Compared with the external oblique spraying method used in general internal grinding, this approach provides a smoother and more uniform electrolyte, effectively reducing its splash.

### 3.3. Structural Design of the Ultrasonic Vibration System

**Figure 5:**
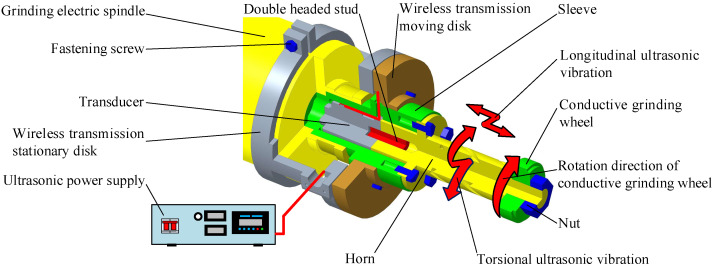
Schematic diagram of the ultrasonic vibration system.

In the special machine tool for ICUAECG, the ultrasonic vibration system is used to convert the ultrasonic electrical signal and energy output by the ultrasonic power supply into the ultrasonic mechanical vibration of the conductive grinding wheel. It is mainly composed of a wireless transmission disk, sleeve, transducer, and horn. Its structure is shown in Figure 5.

The wireless transmission stationary disk is fixed on the housing of the grinding electric spindle through fastening screws, and the sleeve is installed on the rotor of the grinding electric spindle. The wireless transmission moving disk is installed on the sleeve by screws, and the end clearance between the wireless transmission moving disc and the stationary disk is guaranteed to be 0.5 mm. The horn and the transducer are installed together through a double-headed stud and are installed in the sleeve through the flange plate on the horn. The conductive grinding wheel is installed on the horn and fastened with a nut.

In the ICUAECG process, the sleeve, wireless transmission moving disk, horn, transducer, conductive grinding wheel, and rotor of the grinding electric spindle rotate at high speed together, while the wireless transmission stationary disk is stationary. The ultrasonic electrical signal and energy output by the ultrasonic power supply are transmitted from the wireless transmission stationary disk to the moving disk through the role of the ferrite and induction coil in the stationary disk and moving disk and then transferred to the piezoelectric transducer through the wire, shown by the solid red line in Figure 5. The transducer converts the ultrasonic electrical signal and energy into longitudinal ultrasonic mechanical vibration. It then further increases the amplitude of the ultrasonic vibration through the energy accumulation of the horn, which is transmitted to the conductive grinding wheel. The role of the spiral groove on the horn is to convert part of the longitudinal ultrasonic vibration into torsional vibration.

**Figure 6:**
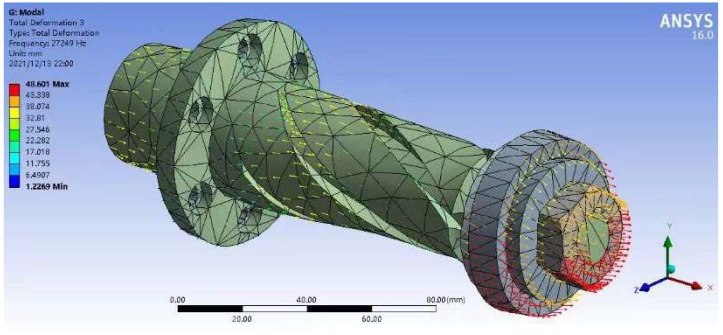
Finite element simulation analysis of horn vibration.

In the ultrasonic vibration system, the function of the horn is to improve the amplitude of the ultrasonic vibration by energy accumulation and transform part of the longitudinal vibration into torsional vibration. The shape and dimension of the horn have a crucial influence on the performance of ultrasonic vibration. In ultrasonic-assisted machining, to give full play to the energy accumulation and amplitude increase in the horn and make the tool obtain a good vibration effect, it is usually designed as a shape with a gradually reduced cross section [3,4,21,22,23,24]. Common horn shapes include conical, exponential, stepped and catenary [3,4,25]. To facilitate processing, the conical horn is used in this study. Local resonance theory [26,27] is used to design and calculate the horn, and finite element analysis software simulates the vibration mode of the horn and harmonic response, as shown in Figure 6. After the horn was manufactured, it was installed in the ultrasonic vibration system of the machine tool, and the conductive grinding wheel was installed. The resonant frequency was measured by measuring the ultrasonic vibration amplitude of the conductive grinding wheel with a laser displacement sensor under no-load conditions.

The simulation and measurement results show that the design of the horn meets the requirements of the ICUAECG. In contrast to previous UAECG studies, which used the ultrasonic electric spindle as the ultrasonic energy input carrier, the proposed special machine tool separates the grinding electric spindle from the ultrasonic vibration system, which has the advantages of low cost and high output ultrasonic power and can provide the ultrasonic vibration of larger grinding wheels.

During the machining of ICUAEG, the ultrasonic vibration is applied to the high-speed rotating conductive grinding wheel, the machining area is in a narrow space, and the electrolyte is also sprayed on the machining area. It is difficult to measure and monitor the ultrasonic vibration frequency and amplitude of the conductive grinding wheel during the machining. Therefore, to simplify the design and manufacturing, the ICUAECG machine tool in the present study adopts an open-loop control for ultrasonic vibration.

### 3.4. On-Machine Dressing Device of the Conductive Grinding Wheel

In ICUAECG, the most used conductive grinding wheel is a metal-bonded sintered one. During machining, the mechanical wear of abrasive particles, chemical corrosion of the metal bonds, and discharge sparks on the surface of the grinding wheel will cause the conductive grinding wheel to wear [6,28,29]. Therefore, to ensure the machining efficiency, accuracy, and surface quality of the ICUAECG, it is necessary to consider the on-machine dressing device of the conductive grinding wheel in the design of the special machine tool. The dressing of the grinding wheel includes two steps: truing and dressing. Truing mainly ensures the grinding wheel accuracy meeting the requirements of shape and profile through the crushing of abrasive particles and the fracture of bonds and ensures that the surface abrasive particles have an excellent agreed height. The dressing is mainly used to remove the bond between the abrasive grains so that the abrasive grains protrude from the surface of the bond, forming a grinding edge with a certain height and a specific chip space. The truing and dressing of the grinding wheel can be carried out simultaneously or step by step [30].

In ICUAECG, when the conductive grinding wheel is fed longitudinally, the standard dressing method of the conductive grinding wheel is to carry out the truing and dressing step by step. First, the conductive grinding wheel is mechanically trued with a diamond roller or SiC grinding wheel. Then, the workpiece is installed on the chuck of the machine tool and connected to the negative pole of the DC power supply. The grinding wheel is connected to the positive pole. The electrochemical dressing of the conductive grinding wheel is carried out using the reverse electrolysis method [31]. This dressing method is convenient. Therefore, in the special machine tool for ICUAECG, the roller truing device of the conductive grinding wheel is equipped.

**Figure 7:**
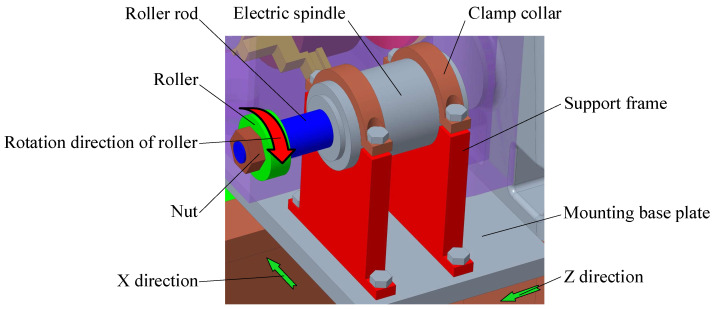
Schematic diagram of the roller truing device of the conductive grinding wheel.

As shown in Figure 7, the roller truing device of the conductive grinding wheel is mainly composed of a mounting base plate, support frame, clamp collar, electric spindle, roller rod, and roller.

During the assembly of the support frame and electric spindle, it should be ensured that the electric spindle and the axes of the roller rod are parallel to the installation axis of the grinding electric spindle. The purpose is to provide the cylindricity of the conductive grinding wheel after truing. During the truing of the conductive grinding wheel, the electric spindle drives the roller rod and roller to rotate. At the same time, under the control of the CNC system, the grinding electric spindle drives the conductive grinding wheel to rotate at a high speed and produces axial and radial feeds along the X- and Z-directions. After the conductive grinding wheel is trued, the reverse electrolysis method can be used for electrochemical dressing.

**Figure 8:**
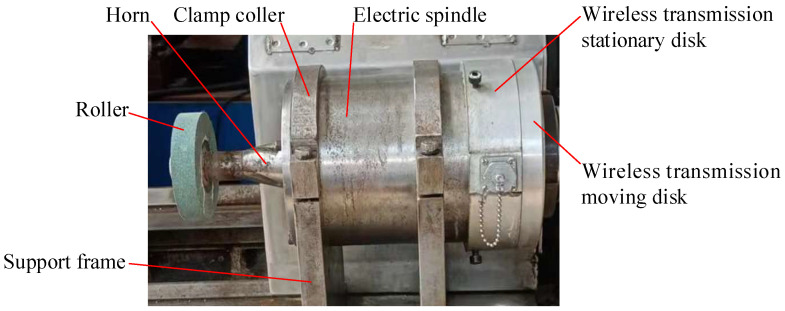
Physical object of the on-machine dressing device of the conductive grinding wheel after adding the ultrasonic vibration system.

In recent decades, ultrasonic vibration dressing technology [31,32,33] on the grinding wheel has developed rapidly. For ICUECG machining, ultrasonic-assisted dressing technology can be applied to the truing of the conductive grinding wheel. During the ultrasonic-assisted truing of the conductive grinding wheel, ultrasonic vibration is applied to the roller to improve the truing quality of the conductive grinding wheel. The physical object of the on-machine dressing device of the conductive grinding wheel after adding the ultrasonic vibration system is shown in Figure 8.

When the ICUAECG adopts radial-feed form grinding, the conductive grinding wheel feeds along the transverse direction during machining. In this case, the EDM dressing can be used. Therefore, the special machine tool is also equipped with an EDM dressing device. The EDM dressing device mainly comprises a dressing electrode, a working-fluid supply, and a circulating system. In the process of EDM dressing, the electrolytic DC power supply can be used as the discharge spark generator power supply; the conductive grinding wheel is connected to the positive pole, and the dressing electrode is connected to the negative pole [34]. Then, the pulse voltage is applied, and the conductive grinding wheel is dressed with the discharge spark generated between the conductive grinding wheel and the dressing electrode.

### 3.5. Grinding Electric Spindle Mounting and Fixing Device

The grinding electric spindle and its mounting device are the key parts of the special machine tool for ICUAECG. Its role is to drive the conductive grinding wheel to rotate at high speed and move in the Z-and X-directions under the control of the CNC system to achieve the feed in the process of ICUAECG and conductive grinding wheel dressing. The grinding electric spindle mounting and fixing device should meet the following requirements: (1) the axis of the grinding electric spindle and the axis of the hollow spindle of the machine tool should have good parallelism to ensure that the cylindricity of the workpiece inner circular surface after ICUAECG is good; (2) the mechanism should have high motion accuracy along the X- and Z-directions; and (3) the grinding electric spindle should be insulated from the machine bed.

**Figure 9:**
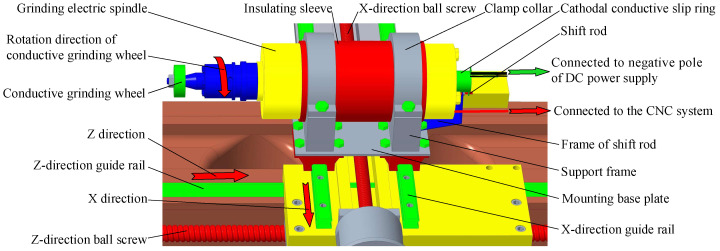
Schematic diagram of the grinding electric spindle mounting and fixing device.

According to the above requirements, the structural design of the grinding electric spindle mounting and fixing device is shown in Figure 9. The device mainly consists of a mounting base plate, support frame, grinding electric spindle, insulating sleeve, clamp collar, cathodal conductive slip ring, X-direction and Z-direction guide rails, and X-direction and Z-direction ball screws. The nominal size of the Z-direction ball screw is 40 mm × 10 mm. The nominal size of the X-direction ball screw is 25 mm × 5 mm. Both adopt a grinding lead screw with an accuracy grade of C7 manufactured by TBI Company. The X-direction guide rail adopts the linear guide rail with the nominal model of SBI30 manufactured by the SBC Company of South Korea. Its surface is subject to an oxidation treatment to ensure its rust resistance. The Z-direction guide rail adopts the V-shaped guide rail provided by the machine bed. An organ shield should protect the X-direction guide rail and ball screw after assembly, and a telescopic cylindrical shield should protect the Z-direction ball screw to prevent corrosion and damage of the guide rail and ball screw caused by electrolyte splash during machining. During the assembly process, the parallelism of the grinding electric spindle and the Z-direction of the machine bed are aligned within 0.01 mm using a dial indicator. The insulating sleeve ensures good insulation between the grinding electric spindle and the machine bed. The cathodal conductive slip ring is installed at the protruding part of the tail of the grinding electric spindle rotor, and the inner ring wire of the cathodal conductive slip ring is connected and fixed on it.

During the ICUAECG, under the control of the NC system, the grinding electric spindle can move along the X- and Z-directions for the machining feed. The conductive grinding wheel rotates at high speed, driven by the rotor of the grinding electric spindle. The inner ring wires of the cathodal conductive slip ring rotate with the rotor of the grinding electric spindle, and the outer ring wires remain stationary under the action of the shift rod. The negative pole of the DC power supply is connected with the conductive grinding wheel through the outer ring wires, the inner ring wires, the grinding electric spindle rotor, and the grinding wheel rod.

## 4. Main Technical Performances of the Special Machine Tool

### 4.1. Physical Object and Main Technical Performances

**Figure 10:**
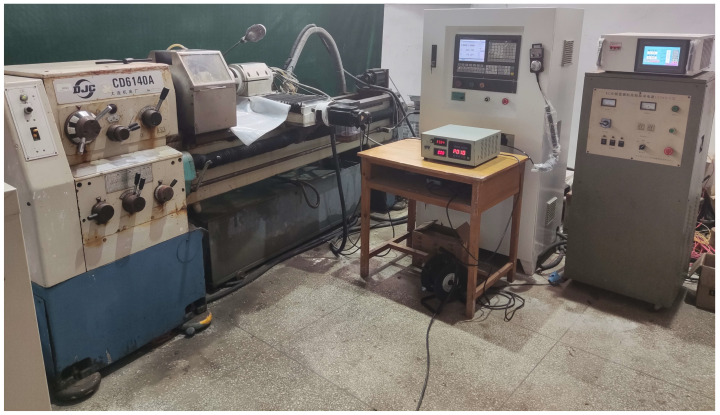
The special machine tool for ICUAECG.

The realized scheme of the special machine tool for ICUAECG is depicted in Figure 10. The machine bed was transformed from a CD6140A lathe. The rapid traverse apron and the saddle of the original lathe were removed, and the ball screw drive mechanism and grinding electric spindle were installed. The carriage and tool rest of the original lathe were removed. The workpiece circumferential feed speed, conductive grinding wheel speed, and axial and radial feed speeds were controlled by the GSK980TDC CNC system. A 150KG24Z10 grinding electric spindle was adopted for machining, with a rated power of 10 kW, working speed of 2000~24,000 r/min, and end-face runout of no more than 1 μm. The radial runout did not exceed 2 μm. The vibration value was not greater than 0.6 mm/s. The HNZM-500 DC power supply had an output voltage of 0~200 V and an output current of 0~50 A. It could adopt two working modes: constant voltage and constant current.

The main technical parameters of the special machine tool are listed in Table 1.

### 4.2. Analysis of Machine Tool Stiffness

The rigidity of the machine tool has an important influence on machining errors. In general, there are two categories of machining errors: quasi-static errors and dynamic errors. The quasi-static mechanical errors are the ones present in the machine, fixturing, tooling, and workpiece that occur relatively slowly, which means their frequency is much lower than the bandwidth of axes on the machine that could be used to correct them. Sources of this type of errors include geometric errors, kinematic errors, external load-induced errors, machine-assembly errors, thermal expansion errors, material-instability errors, and instrumentation errors [35]. The quasi-static errors, specifically cutting force-produced errors, account for approximately 75% of the total errors of the machine [36]. During the manufacture of the special machine tool for ICUAECG, the grinding electric spindle was calibrated to ensure that its vibration value was less than 0.6 mm/s in high-speed rotation, and the conductive grinding wheel was also calibrated to ensure its good dynamic balance feature. Therefore, we focused on the quasi-static errors of the special machine tool.

For the special machine tool for ICUAECG in the present study, the machine bed was transformed from a CD6140A lathe. The grinding force of ICUECG is significantly less than that of ordinary grinding, and much less than that of turning. The machine tool deformation caused by grinding force in ICUECG processing is far less than that in turning. In addition, during the transformation of the machine bed, the rapid traverse apron and the saddle of the original lathe were removed and replaced by the ball screw drive mechanism and the grinding electric spindle mounting and fixing device, respectively. The weight of the ball screw transmission mechanism was less than the rapid traverse apron. The weight of the grinding electric spindle was less than the saddle. Therefore, the deformation of the machine bed caused by the displacement of moving parts was significantly less than that in the turning process of the original lathe. Therefore, the machine stiffness of the special machine tool could meet the requirements of ICPECG machining accuracy.

In a future study, the frequency domain analysis method proposed by Leonesio et al. [37] could be used to further study the equivalent stiffness and damping in the machining of ICPECG special machine tools, to provide a basis for avoiding grinding chatter in the processing.

## 5. Machining Experiments Using the Special Machine Tool

To verify the operability of the special machine tool for ICUAECG, the ICUAECG experiment of the bearing ring was carried out with the special machine tool. The workpiece material was GCr15 bearing steel in the quenched state, and the diameter of the inner hole before machining was 100 mm. A CBN conductive grinding wheel sintered with a bronze bond, a 90# abrasive grain size and an outer diameter of 60 mm was used for machining. The abrasive concentration was 150%, and electrolyte used for machining was a NaNO_3_ aqueous solution with a 20% mass concentration.

### 5.1. Dressing of the Conductive Grinding Wheel

The conductive grinding wheel was dressed before machining to ensure efficiency and accuracy. First, an abrasive SiC wheel was used as a roller to true the conductive grinding wheel. The outside diameter of the abrasive SiC wheel was 100 mm, and its particle size was 90#. The longitudinal feeding mode was adopted in the truing process. The truing ensured that the radial runout of the outer circle of the conductive grinding wheel was at most 0.02 mm. Then, the conductive grinding wheel was connected to the positive pole of the DC power supply through the cathodal conductive slip ring, and the workpiece was connected to the negative pole of the DC power supply through the anodic conductive slip ring so that the conductive grinding wheel was dressed via the reverse electrolysis method. The preset process parameters of truing and dressing are listed in Table 2.

**Figure 11:**
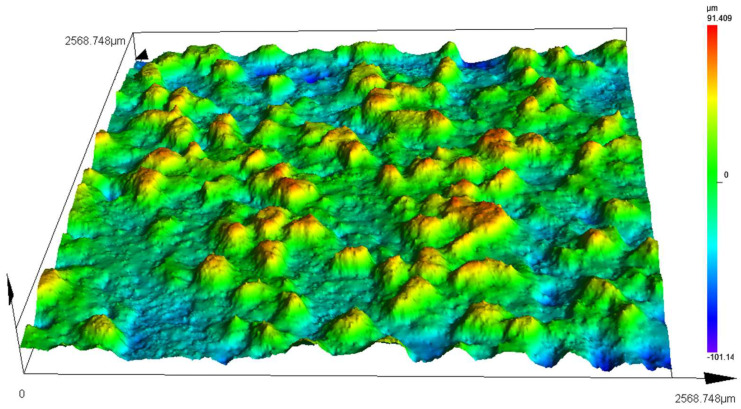
Microtopography of surface abrasive particles of the conductive grinding wheel after dressing.

After the conductive grinding wheel was dressed, an OLS5100 Olympus laser confocal microscope was used to observe the micromorphology of the surface abrasive particles, as shown in Figure 11.

The measured protrusion height of the abrasive particles ranged from 0.04 to 0.09 mm, with an average value of 0.063 mm. The average size of 90 # abrasive particles is about 0.165 mm. After the conductive grinding wheel was dressed, the average protrusion height of the abrasive grain was close to 1/3 of its average size, fully satisfying the grinding requirements [38].

### 5.2. Machining Experiment of ICUAECG

After the conductive grinding wheel was dressed, the workpiece was connected to the positive pole of the DC power supply, and the conductive grinding wheel was connected to the negative pole. The ICUAECG experiment was carried out, with process parameters shown in Table 3. In the machining, the radial-feed form grinding was adopted, with the conductive grinding wheel feeding along the transverse direction.

**Figure 12:**
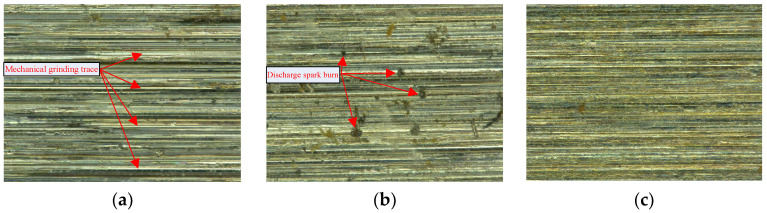
Comparison of three machined surface topographies: (**a**) internal cylindrical ordinary grinding; (**b**) internal cylindrical ECG; (**c**) ICUAECG.

The machining experiments were also carried out for (i) the internal cylindrical ECG with disconnected ultrasonic power supply and (ii) the internal cylindrical ordinary grinding with disconnected ultrasonic and DC power supplies. The respective process parameters are specified in Table 2. The machined surfaces of ordinary grinding, ECG, and ICUAECG were examined via a super-depth field microscope. The comparison of the surface topographies of the machined surfaces magnified 300 times under the three processing conditions is shown in Figure 12.

It can be seen from Figure 12 that the mechanical grinding traces of the machined surface of the internal cylindrical ECG were less pronounced than those of the internal cylindrical ordinary grinding. Still, the machined surface generated discharge spark burns due to the unstable machining and electrode short circuit [6,39]. On the machined surface of ICUAECG, the discharge spark burn was effectively avoided. This is because the ultrasonic vibration made the flow field of the electrolyte more uniform than that of ECG, and the electrolytic products were more conducive to discharge from the machining area, leading to the practical improvement of the stability of ECG.

**Figure 13:**
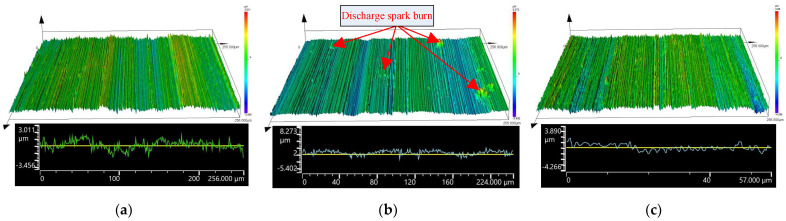
Comparison of machined-surface roughness under the three processing conditions: (**a**) internal cylindrical ordinary grinding; (**b**) internal cylindrical ECG; (**c**) ICUAECG.

The comparison of the machined-surface roughness observed by the OLS5100 Olympus laser confocal microscope under the three processing conditions is shown in Figure 13.

It can be observed from Figure 13 that the surface roughness of ordinary grinding was R_Z_0.87 μm, the surface roughness of ECG was R_Z_0.64 μm, and the surface roughness of ICUAECG was R_Z_0.58 μm. The discharge spark burn was also observed on the surface machined by ECG. The surface roughness value of ICUAECG was the minimum, and the discharge spark burn was avoided. Thus, ICUAECG manifested obvious technical advantages over ordinary grinding and ECG.

## 6. Conclusions

This study proposed a new structural design for a special machine tool for internal cylindrical ultrasonic-assisted electrochemical grinding (ICUAECG). According to the characteristics of ICUAECG, the structure of the special machine tool was designed. According to the proposed design scheme, a special machine tool was manufactured, and conductive grinding wheel dressing and ICUAECG experiments were carried out using the proposed special machine tool. The results obtained made it possible to draw the following conclusions:

1. In the structural design of the special machine tool for ICUAECG, the proposed workpiece clamping and rotating device combined the functions of rotating clamping, electrolyte supply and spraying, workpiece connection, body insulation, etc. It effectively used the internal space of the hollow spindle, reduced the volume of the external suspension devices and the machine tool profile, ensured the appropriate spraying direction of the electrolyte and the uniformity of the electrolytic flow field, reducing the electrolyte splash.
2. The proposed ultrasonic vibration system structural design was based on using an ordinary grinding electric spindle, which effectively reduced the manufacturing cost of the special machine tool, improved the output ultrasonic vibration power, and made the system applicable to the ICUAECG processing of large conductive grinding wheels.
3. The movement of the grinding electric spindle mounting and fixing device was transmitted through the high-precision ball screw and the linear guide rail and controlled by the CNC system, ensuring travel accuracy during ICUAECG, and thus guaranteeing high machining accuracy and surface quality.
4. The experiments of the conductive grinding wheel dressing and ICUAECG were performed, proving that, compared with internal cylindrical ordinary grinding and internal cylindrical ECG, ICUAECG more effectively reduced the mechanical grinding traces of the machined surface, reduced the surface roughness, effectively avoided the discharge spark burns in the process of ECG, and significantly improved the machined-surface quality. The developed special machine tool met the requirements of conductive grinding wheel dressing and ICUAECG.

## Figures and Tables

**Table 1:** Main technical parameters of the special machine tool for ICUAECG.

Technical Parameter	Value
Size range of machined inner hole	30~150 mm
Maximum grinding length	100 mm
Output power of grinding electric spindle	10 kW
Rotation speed of grinding electric spindle	2000~24,000 r/min
Allowable grinding wheel diameter	20~100 mm
Workpiece speed range	0~200 r/min
DC power supply voltage output range	0~200 V
DC power current range	0~50 A
DC power duty cycle range	0~100%
Frequency range of ultrasonic vibration	20~35 kHz
Ultrasonic vibration amplitude	0~2.5 μm

**Table 2:** The process parameters of conductive grinding wheel truing and dressing.

	Process Parameter	Value
Truing	Rotating speed of the conductive grinding wheel	3000 r/min
	Rotating speed of the roller	1000 r/min
	Single trip feed depth	0.05 mm
Dressing	DC power supply voltage	10 V
	Rotating speed of the conductive grinding wheel	6000 r/min
	Rotating speed of the workpiece	35 r/min
	Clearance between grinding wheel and workpiece	0.05 mm
	Flow of electrolyte	9.5 L/min
	Duration	2 min

**Table 3:** Process parameters of the ICUAECG experiment.

Technical Parameter	Value
Frequency of ultrasonic vibration	31.3 kHz
Amplitude of ultrasonic vibration	0.0018 mm
DC power supply voltage	10 V
Rotating speed of the conductive grinding wheel	6000 r/min
Rotating speed of the workpiece	35 r/min
Radial feed rate	0.2 mm/min
Flow of electrolyte	9.5 L/min
Machining allowance	0.05 mm

## Data Availability

Not applicable.
